# A new species and a new species record of Orbiniidae Hartman, 1942 (Annelida, Polychaeta) from China

**DOI:** 10.3897/zookeys.1068.71925

**Published:** 2021-11-02

**Authors:** Yue Sun, Lei Yu, Xinzheng Li

**Affiliations:** 1 School of wetland, Jiangsu Provincial Key Laboratory of Coastal Wetland Bioresources and Environmental Protection, Jiangsu Key Laboratory for Bioresources of Saline Soils, Yancheng Teachers University, Yancheng 224051, China Yancheng Teachers University Yancheng China; 2 Institute of Oceanology, Chinese Academy of Sciences, Qingdao 266071, China Institute of Oceanology, Chinese Academy of Sciences Qingdao China; 3 Center for Ocean Mega-Science, Chinese Academy of Sciences, Qingdao 266071, China Center for Ocean Mega-Science, Chinese Academy of Sciences Qingdao China; 4 University of Chinese Academy of Sciences, Beijing 200049, China University of Chinese Academy of Sciences Beijing China; 5 Laboratory for Marine Biology and Biotechnology, Qingdao National Laboratory for Marine Science and Technology, Qingdao, China Laboratory for Marine Biology and Biotechnology, Qingdao National Laboratory for Marine Science and Technology Qingdao China

**Keywords:** China Seas, new record, *Phylo*, taxonomy

## Abstract

A new species of the orbiniid genus *Phylo* Kinberg, 1866, *P.heterochaetus***sp. nov.**, is described based on material collected from the northern Yellow Sea, China. This is the thirteenth species in *Phylo*. The new species can be easily identified by the combination of the following characters: anterior thorax with 13 chaetigers, interramal cirri absent, anterior thoracic neuropodia with 4 or 5 rows of uncini, intermixed with a few subuluncini in the first 1 or 2 rows, and a ventral fringe of numerous stomach papillae present on chaetigers 12–24. *Phylofimbriata* is recorded for the first time from China seas.

## Introduction

*Phylo* Kinberg, 1866 belongs to Orbiniidae Hartman, 1942 and differs from the other genera of that family in having species with modified spines on posterior thoracic chaetigers. Although species of *Phylo* are closely related to *Orbinia* species based on molecular analyses (Bleidorn 2005; Bleidorn et al. 2009), *Phylo* is retained as a genus because the presence of posterior thoracic modified spines is a distinctive and easily recognized characteristic for species of *Phylo* (Blake 2017). Hartman (1957) reviewed this genus and described four species in detail, including the type species, *P.felix* Kinberg, 1866. Subsequently, Day (1961, 1977), Mohammad (1980), Hartmann-Schröder and Rosenfeld (1990), and Blake (2017, 2020, 2021) described additional *Phylo* species. According to this literature, 14 species of *Phylo* are currently recognized and considered valid (Blake 2021; Read and Fauchald 2021).

The genus *Phylo* is characterized as follows: branchiae first present from chaetiger 5–7; thoracic neuropodia fringed, with several postchaetal lobes; posterior thorax and anterior abdominal chaetigers with subpodial lobes, usually forming a ventral fringe; thoracic neurochaetae including blunt uncini or subuluncini and crenulated capillaries; posterior thoracic chaetigers with modified spines.

According to Liu (2008), four species have been documented from Chinese waters: *Phylofelix*, *Phyloornatus* (Verrill, 1873), *Phylokupfferi* (Ehlers, 1874), and *Phylonudus* (Moore, 1911). We checked specimens previously identified as *P.felix* deposited in the Marine Biological Museum of the Chinese Academy of Science and found the report of the species from Chinese waters to be a misidentification. These specimens were identified as a new species, which is described and illustrated in this study. Additionally, we describe and illustrate *Phylofimbriata* based on specimens collected from the East China Sea. This is the first record of *P.fimbriata* from Chinese waters.

## Material and methods

Material checked in this study was collected from the northern Yellow Sea and East China Sea and deposited in the Marine Biological Museum of Chinese Academy of Science, with a few specimens retained by the present college of the first author (Yancheng Teachers University, YCTU). All the specimens were preserved in 75% ethanol solution. Detailed morphological structures were examined under a Zeiss Stemi 2000-C stereomicroscope. Photography and line drawings were made using an AxioCam MRc 5 digital camera attached to a stereomicroscope and a compound microscope. Specimens were stained with rose bengal to observe details of parapodial lobes and subpodial lobes. For SEM observations, selected parapodia were detached from the specimens, rinsed in absolute ethanol, dehydrated, coated in gold, observed, and photographed using a scanning electron microscope.

The following abbreviations are used:

**MBM** Marine Biological Museum;

**YCTU** Yancheng Teachers University;

**spec** specimen;

**Sta.** station;

**ECS** East China Sea;

**nYS** Northern Yellow Sea;

**SEM** scanning electron microscope.

## Taxonomy

### Family Orbiniidae Hartman, 1942

**Subfamily Orbiniinae Hartman, 1942**

#### Genus Phylo

Taxon classificationAnimaliaScolecidaOrbiniidae

Kinberg, 1866

C92BD80B-E819-50D1-8337-3542C90E181D

##### Type species.

*Phylofelix* Kinberg, 1866

#### Phylo heterochaetus sp. nov.

Taxon classificationAnimaliaScolecidaOrbiniidae

A2E7DB98-6B78-5109-BE12-E30E45C24BEC

http://zoobank.org/AAAAD262-9B46-4B96-BD38-F6FBF8F7D5A3

Figures 1 , 2 , 3

##### Material examined.

***Holotype***: MBM286984: nYS, Sta. 3875–04, 38°45'N, 123°30'E, 64 m, 16 Dec. 2015. ***Paratype***: MBM286985: nYS, Sta. 16, 39°07'N, 122°54'E, 31 m, 18 Dec. 2016. MBM286986: nYS, Sta. C3, 39°00'N, 122°55'E, 45 meters, 18 Dec. 2016. Sta. MBM286987: nYS, Sta. A1, 39°05'N, 122°35'E, 36 m, 18 Dec. 2016. MBM023255: nYS, Sta. 2049, 122°45'N, 38°09'E, 51 m, coll. Mu Chen. 11 Jul. 1959.

##### Additional material.

MBM023221: nYS, Sta. 2056, 38°30'N, 123°30'E, 63 m, Mu Chen. 15 Apr. 1959. MBM009975: nYS, Sta. 2057, 38°00'N, 123°30'E, 70.5 m, coll. Yuheng Cui. 1 Oct. 1958. YCTU000004: nYS, Sta. A3, 39°00'N, 122°35'E, 35 m, 18 Dec. 2016. YCTU000005: nYS, Sta. 48, 39°16'N, 123°18'E, 37 m, 18 Dec. 2016.

##### Description.

All specimens incomplete, posterior end missing, holotype with 60 chaetigers, 34 mm long and 5 mm wide. Body elongate, about same width throughout; thorax depressed, abdomen cylindrical.

**Figure 1.**
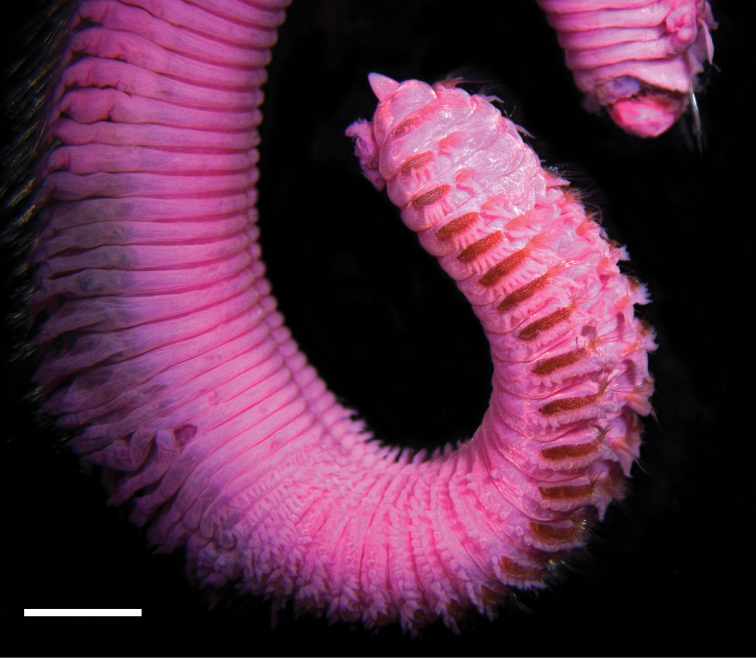
Anterior end of *Phyloheterochaetus* sp. nov. Scale bar: 2 mm.

Prostomium short, conical, tapering to rounded tip; eyepots absent, nuchal organs narrow groove at junction with peristomium (Fig. 1). Peristomium a narrow asetigerous segment, distinctly separated from prostomium and chaetiger 1; mouth with anterior oral lip arising from posterior margin of peristomium, posterior oral lip from anterior margin of chaetiger 2. Holotype with proboscis everted, consisting of two large, inflated lobes (Fig. 1).

**Figure 2.**
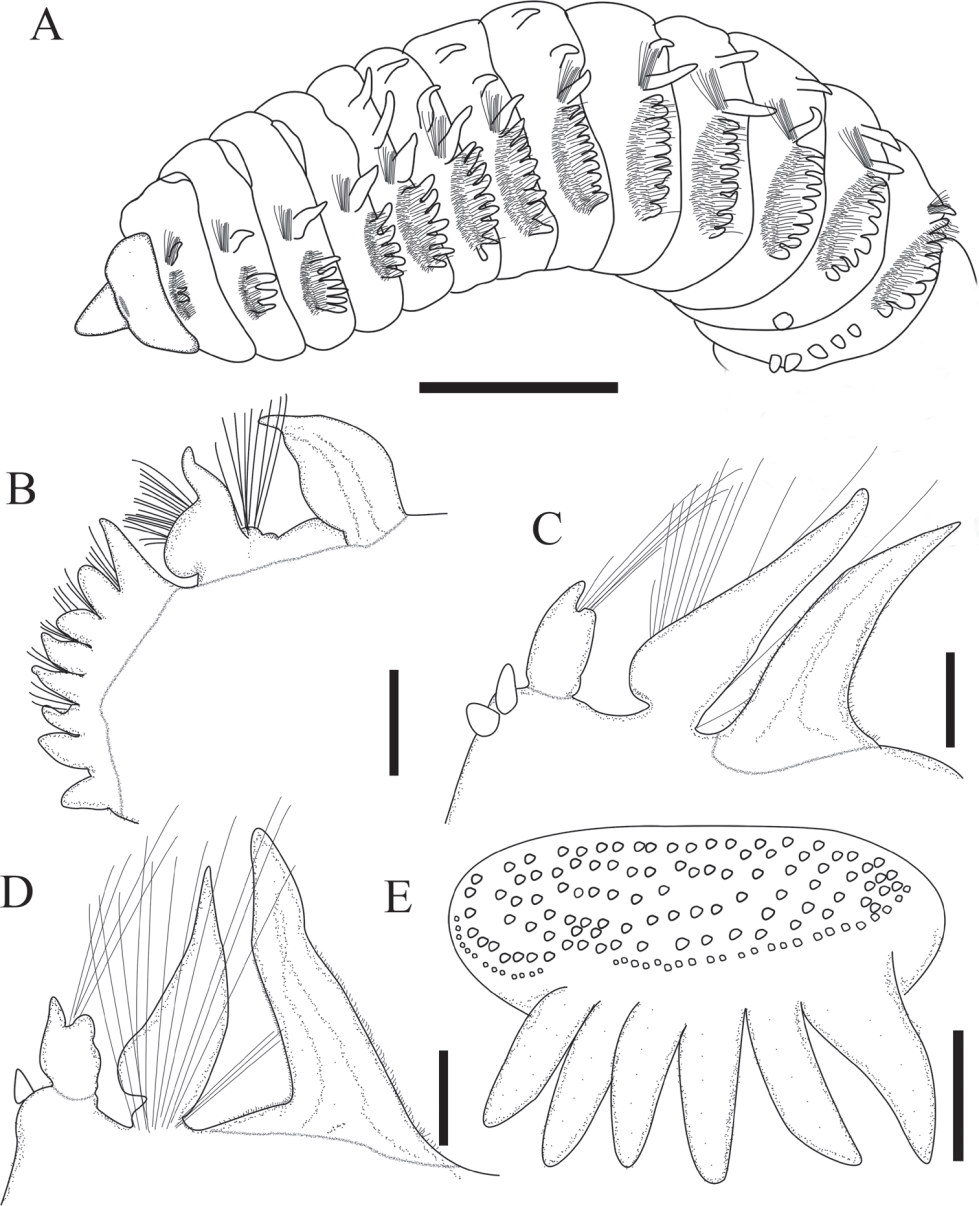
*Phyloheterochaetus* sp. nov. **A** anterior body in lateral view **B** parapodium of chaetiger 7 **C** parapodium of anterior abdominal chaetiger **D** parapodium of chaetiger 62 **E** diagram showing arrangement of anterior thoracic neurochaetae. Scale bars: 2 mm (**A**); 400 μm (**B–D**); 200 μm (**E**).

Holotype with 21 thoracic chaetigers, divided into anterior and posterior sections: anterior thorax with 13 chaetigers (Figs 1, 2A), posterior thorax with modified spines beginning on chaetiger 14 continuing over 8 chaetigers. Intersegmental annulations first present from chaetiger 1, well developed (Figs 1, 2A). Thoracic notopodial postchaetal lobe arising from narrow base, broadly triangular, narrowing apically (Fig. 2B), becoming long and narrow in abdominal chaetigers (Fig. 2C, D). Thoracic neuropodia thick, elongate, fringed, bearing rows of uncini and several capillaries. First chaetiger with 2 rounded postchaetal lobe, increasing to 13–14 on middle thoracic chaetigers (Fig. 2A), then decreasing to 6 or 7 on last thoracic chaetigers. Subpodial lobes or stomach papillae from chaetiger 12, numbering 1 at first, then extending to ventral midline on chaetigers 18–25, then abruptly absent (Figs 1, 2A). Abdominal neuropodia bilobed, with inner lobe blunt, outer lobe cirriform (Fig. 2C, D). First abdominal chaetiger with 6 extra lobes or ventral cirri ventral to neuropodium, then reduced to 2 on following 3 chaetigers and 1 or 2 on subsequent chaetigers (Fig. 2C, D). Interramal cirrus absent.

Branchiae from chaetiger 5, on anterior chaetigers short, triangular, tapering to narrow papillate rounded apex (Fig. 2B), then becoming narrower and longer, leaf-like and ciliated (Fig. 2C, D).

**Figure 3.**
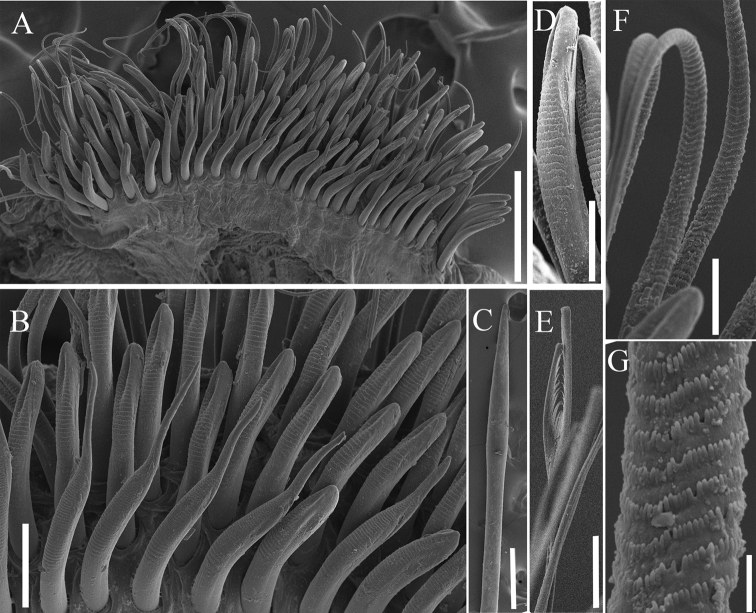
SEM pictures of *Phyloheterochaetus* sp. nov. **A** neurochaetae of thorax **B** uncini and subuluncini **C** modified spines **D** uncini of last rows of thoracic neuropodia **E** furcate chaeta **F, G** crenulate capillaries. Scale bars: 250 μm (**C**); 200 μm (**A, E, F**); 50 μm (**B**); 30 μm (**G**); 10 μm (**D**).

Thoracic notopodia with fascicles of crenulated capillaries. Abdominal notopodia with few long, thin capillaries, 2–4 furcate chaetae and 3–5 imbedded aciculae, furcate chaetae with unequal tynes, each tyne with fine needles directed medially, shaft with transverse rows of barbs (Fig. 3E).

Anterior thoracic neuropodia with 4 rows and 1 short posterior rows of yellow uncini, and posterior row of crenulate capillaries (Figs 2E, 3A, B, E, F). Anterior 2 rows mixed with 10–12 subuluncini, last row with 7–9 uncini, curved ventrally to anterior rows (Fig. 3A, B). Uncini of first 1–4 rows short, with blunt-tipped apex, with 10–18 transverse rows of blunt barbs on convex side, with conspicuous groove apically (Fig. 3B, D); uncini of last row longer than anterior ones, only located in ventral side of neuropodium (Fig. 3A); subuluncini resemble uncini, but with distally pointed tip, with more than 30 rows of blunt barbs, surrounded by sheath (Fig. 3B). Posterior thoracic neuropodia with 4–7 modified spines (Fig. 3C), single short row of uncini and 1 or 2 rows crenulate capillaries. Modified spines large, hastate, superior one projecting from neuropodium, with glandular pouch (Fig. 3C). Abdominal neurochaetae including 4–6 thin capillaries and single acicula, thin and imbedded.

Anterior thoracic neuropodia with 5 or 6 rows of yellow uncini and posterior row of crenulate capillaries (Figs 2E, 3A, B). Anterior 2 rows mixed with 10–12 subuluncini, last row with 7–9 uncini, curved ventrally to anterior rows (Fig. 3A, B). Uncini of anterior 4 or 5 rows short, with blunt-tipped apex, with 10–18 transverse rows of blunt barbs on convex side, with conspicuous groove apically (Fig. 3B, D); uncini of last row longer than anterior ones, only located in ventral side of neuropodium (Fig. 3A). Subuluncini resemble uncini but with distally pointed tip, with more than 30 rows of blunt barbs, surrounded by sheath (Fig. 3B). Posterior thoracic neuropodia with 4–7 modified spines (Fig. 3C), single short row of uncini and 1 or 2 rows crenulate capillaries. Modified spines large, hastate, superior one projecting from neuropodium (Fig. 3C). Abdominal neurochaetae including 4–6 thin capillaries and single acicula, thin and imbedded.

Pygidium not observed.

##### Variation.

One paratype (MBM286986) with 13 and 14 anterior thoracic chaetigers on left and right side respectively, posterior thorax with 9 and 8 chaetigers, respectively. Holotype and other specimens with 13 anterior thoracic chaetigers, posterior thorax with 4–8.

##### Etymology.

The species is named for the thoracic neuropodia with two kinds of uncini.

##### Type locality.

Northern Yellow Sea, China.

##### Remarks.

This species is unusual in the genus in having anterior thoracic neuropodia with 5 or 6 rows of uncini intermixed with a few subuluncini in the first 1 or 2 rows. *Phyloheterochaetus* sp. nov. is similar with *P.ornatus* (Verrill, 1873) for: 1) anterior thoracic neuropodia with rows of uncini, 2) lacking interramal cirri, 3) anterior thorax with 13 or 14 chaetigers. They can be easily distinguished by 1) species of *P.heterochaetus* sp. nov. with 4–8 posterior thoracic chaetigers, while the latter species with 13 or more chaetigers; 2) species of *P.heterochaetus* sp. nov. with 10 or 11 subuluncini intermixed with first 2 rows uncini, while the latter species lacks subuluncini; 3) modified spines hastate in *P.heterochaetus* sp. nov. and acicular in *P.ornatus*.

#### Phylo fimbriata

Taxon classificationAnimaliaScolecidaOrbiniidae

(Moore, 1903)

6CE87C04-0F9A-5BEE-A185-8F6652DE9309

Figures 4 , 5 , 6

Aricia fimbriata Moore, 1903: 464–467, pl. XXIV, figs 33–35; Okuda 1937: 99–101, figs 1, 2.
Phylo fimbriata : Hartman 1957: 267.

##### Material examined.

YCTU000006: ECS, Sta. 3100–8, 31°00'N, 126°00'E, 51 m, soft mud substrate, Jun. 2014. YCTU000007: ECS, Sta. I3, 33°00'N, 123°00'E, 34 m, soft mud substrate, 20 Sep. 2015. MBM009966: ECS, Sta. 4134, 29°30'N, 123°00'E, 50 m, soft mud substrate, 20 Jan. 1959. MBM 023276: ECS, Sta. 4007, 33°30'N, 122°30'E, 37 m, soft mud substrate, 13 Apr. 1959.

##### Description.

All specimens incomplete, posterior end missing. Body elongate, about same width throughout; thorax depressed, abdominal chaetigers cylindrical.

Prostomium short, conical, tapering to rounded tip; eyepots absent; nuchal organs narrow groove at junction with peristomium. Peristomium an asetigerous segment, distinctly separated from prostomium and chaetiger 1; mouth with anterior oral lip arising from posterior margin of peristomium, posterior oral lip from anterior margin of chaetiger 2. Proboscis everted, consisting of 2 large, inflated lobes.

**Figure 4.**
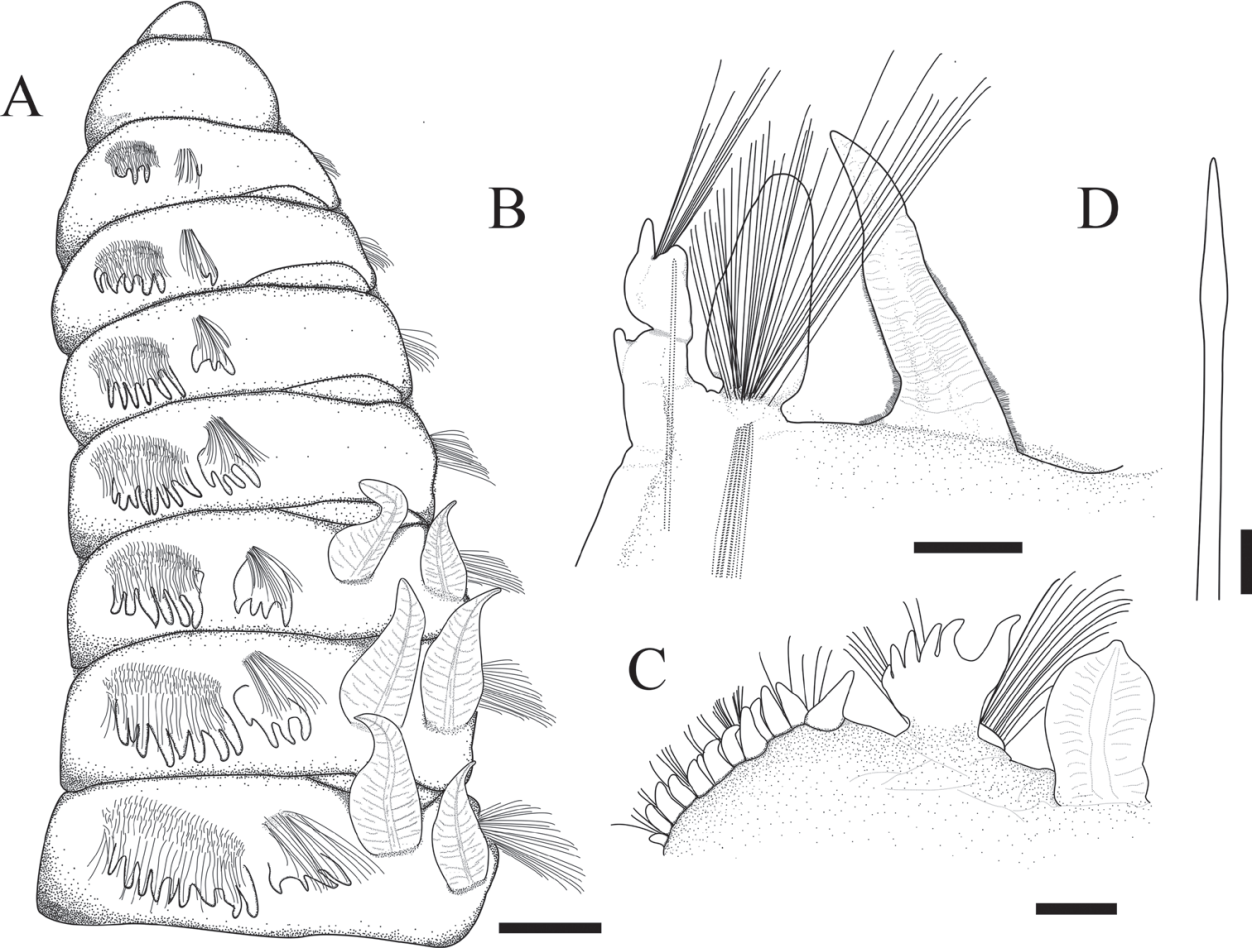
*Phylofimbriata* (Moore, 1903) **A** anterior end, left lateral view **B** parapodium of abdominal chaetigers; **C** parapodium of thoracic chaetigers **D** modified spine from posterior thoracic chaetiger. Scale bars: 1 mm (**A**); 500 μm (**B–D**).

Thorax with 16 chaetigers, divided into anterior and posterior sections: anterior thorax with 12 chaetigers, posterior thorax with modified spines from chaetiger 13 continuing over 4 chaetigers. Intersegmental annulations first present from chaetiger 1, well developed (Fig. 4A).

**Figure 5.**
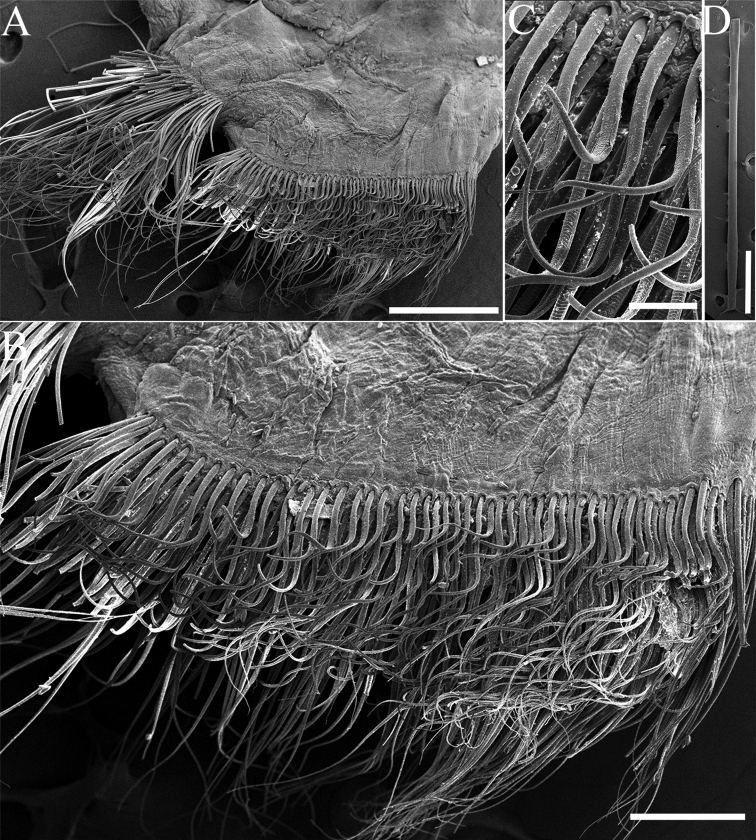
SEM pictures of *Phylofimbriata* (Moore, 1903) **A** anterior thoracic chaetiger, anterior view **B** same, detail, showing arrangement of neurochaeta **C** same, detail, showing neurochaetae **D** modified spines. Scale bars: 5 mm (**A, D**); 200 μm (**B**); , 50 μm (**C, D**).

Both thoracic notopodia and neuropodia with fringed postchaetal lobes; notopodium half as broad as the neuropodium, broadly palmate (Figs 4A, 5A). Notopodia with single conical postchaetal lobe from chaetiger 1, increasing gradually to about 6 lobes on posterior thoracic chaetigers; postchaetal lobes equivalent in size and shape on anterior chaetigers, with inner lobe separate and becoming longer than the outer ones on the posterior chaetigers (Fig. 4A, C). Abdominal notopodial postchaetal lobe arising from narrow base, broadly triangular (Fig. 4B).

**Figure 6.**
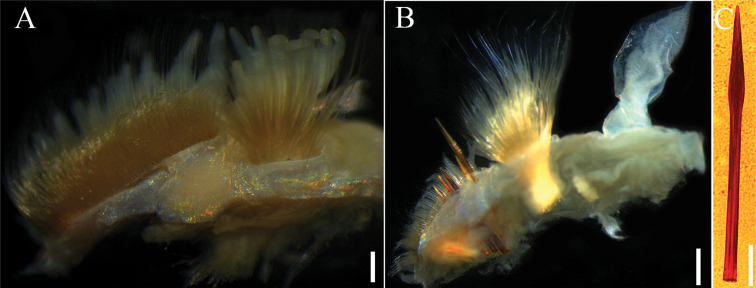
*Phylofimbriata* (Moore, 1903) **A** anterior thoracic chaetiger, anterior view **B** posterior thoracic chaetiger, anterior view **C** modified spines. Scale bars: 0.5 mm (**B, C**); 0.2 mm (**A**).

Thoracic neuropodia thick, elongate, strictly lateral, bearing numerous subuluncini and capillaries (Fig. 4A, C). Neuropodium with 1–3 conical postchaetal lobes from chaetiger 1, increasing gradually to about 15 lobes on chaetigers 9 (Figs 4A, C, 6A). Subpodial lobes or stomach papillae present on chaetiger 17–19, numbering 2 or 3. Abdominal neuropodia bilobed, with inner lobe blunted, outer lobe cirriform (Fig. 4B). First abdominal chaetiger with 3–6 extra lobes or ventral cirri ventral to neuropodium, then reduced to a single cirrus on subsequent chaetigers (Fig. 4B). Interramal cirrus absent.

Branchiae from chaetiger 5, each anterior branchia broad, tapering to narrow rounded tip (Fig. 4C), subsequent branchiae narrower and longer, leaflike and ciliated (Fig. 4B).

Thoracic notopodia with fascicles of crenulated capillaries (Figs 5A, 6A, B). Abdominal notopodia with fewer long, thin capillaries, 3 or 4 furcate chaetae and 4–8 imbedded aciculae (Fig. 4B). Anterior thoracic neuropodia with numerous yellow subuluncini and crenulated capillaries (Figs 5B, 6A). Subuluncini arranged in dense phalanx, with anterior ranks strongly curved, much shorter than posterior ranks (Fig. 5A, B). Subuluncini tapering to narrow pointed tip, covered with sheath, shaft with transverse rows of minute ribs or barbs (Fig. 5C). Posterior thoracic neuropodia with 4 or 5 modified spines, several subuluncini and crenulated capillaries ventral to the modified spines (Fig. 6B). Modified spines large, superior one projecting from neuropodium, with glandular pouch (Figs 4D, 5D, 6B, C). Abdominal neurochaetae including thin capillaries and 1–3 aciculae (Fig. 5B).

Pygidium not observed.

##### Distribution.

East China Sea (China); Suruga Bay, Miyagi Prefecture (Japan).

##### Remarks.

*Phylofimbriata* (Moore, 1903), which was first reported by Moore (1903) from Suruga Bay and North Japan, has fringed postchaetal lobes on the thoracic notopodia unlike most species of *Phylo*. Okuda (1937) redescribed this species based on specimens from Miyagi Prefecture. The morphology of present specimens agrees well with the original description of specimens from Japan.

## Supplementary Material

XML Treatment for Phylo

XML Treatment for Phylo heterochaetus

XML Treatment for Phylo fimbriata
